# The Influence of Environmental Exposure to Heavy Metals on the Occurrence of Selected Elements in the Maxillary Bone

**DOI:** 10.3390/ijms24032552

**Published:** 2023-01-29

**Authors:** Piotr Malara, Maciej Misiołek, Agnieszka Fischer, Beata Malara

**Affiliations:** 1 Department of Maxillofacial Surgery for Children, Chorzow Hospital for Paediatrics and Oncology, 41-500 Chorzów, Poland; 2 Postgraduate Educational Centre of Dentistry DENTARIS, School of Medicine, Katowice Business University, 40-659 Katowice, Poland; 3 Department of Otorhinolaryngology and Oncological Laryngology, Medical University of Silesia, 41-800 Zabrze, Poland; 4 Department of Toxicology and Bioanalysis, Medical University of Silesia, 41-200 Sosnowiec, Poland; 5 Department of Facial Aesthetics and Cosmetology, School of Medicine, Katowice Business University, 40-659 Katowice, Poland

**Keywords:** lead, cadmium, maxilla, bone chemistry, biomonitoring, environmental exposure, atomic absorption spectrometry

## Abstract

The elemental composition of the body’s calcified tissues may reflect the environmental exposure of the population to heavy metals. The aim of the study was to assess whether the elemental composition of the maxillary bone from individuals belonging to a given population reflects the environmental exposure of this population to lead and cadmium. The research material consisted of cortical bone from the anterolateral walls of the maxilla collected from 126 patients during Caldwell–Luc maxillary sinus surgery on residents of two cities differing in terms of the lead and cadmium pollution of the natural environment. The content levels of lead, cadmium, iron, manganese, chromium, copper, and iron were determined by using atomic absorption spectrophotometry. The content levels of lead and cadmium in the samples of the maxillary bones of residents of Bielsko-Biala were 3.26 ± 2.42 µg/g and 0.74 ± 0.38 µg/g, respectively, whereas in the samples from the residents of Katowice, they were 7.66 ± 2.79 µg/g and 1.12 ± 0.08 µg/g, respectively. It was found that the lead and cadmium levels in the maxillary bone corresponded to the environmental exposure to these heavy metals in the place of residence, which was proven here via the example of the residents of two cities with different concentrations of these heavy metals in the air over long time periods. Additionally, higher content levels of essential metals such as manganese, chromium, copper, and iron are characteristic of the maxillary bone samples of residents of the area that is more polluted with heavy metals.

## 1. Introduction

The chemical composition of calcified tissues in living organisms can reflect their health status as well as nutrition and human exposure to chemicals of environmental or occupational origin [1,2,3]. Teeth and bones have been used in forensic medicine for years to identify murder victims and ancient human remains, allowing them to be assigned to specific historical periods in which these organisms developed [4]. There are many studies that have proven that there is a relationship between behavioral deficits in children and the mineralogical composition of their calcified tissues, especially deciduous teeth [5].

Both teeth and bones are examples of calcified tissues, the main building component of which is calcium hydroxyapatite. Although the mineral composition of these tissues is relatively stable, elements from food, air, and water entering living organisms can change the composition of these tissues mainly by substituting calcium ions in hydroxyapatites [6].

For many years, deciduous teeth were mainly used to assess the body’s exposure to heavy metals [7,8,9]. This is due to the relative ease of obtaining this biological material during the period of natural replacement of the dentition [10]. Obtaining this material for research does not raise any ethical concerns. It is also known that permanent teeth can be used to assess environmental exposure to heavy metals, although the availability of this biological material is limited only to impacted teeth that remain in the bone after the period of natural tooth replacement [11] or to carious or broken teeth not suitable for conservative dental treatment [12]. In the latter case, only the roots of these teeth are suitable for the evaluation of the mineralogical structure [13]. It should be noted that various analytical methods can be used to determine the metal content in teeth. In most studies, Laser Ablation Inductively Coupled Plasma Mass Spectrometry (ICP-MS) is used, which is considered less destructive and enables determination of heavy metals in small volumes of several tooth tissues including, for example, enamel [7,8,9]. Flame Atomic Absorption Spectrometry (FAAS), which is used, among other methods, in the research of our team [11,13,14] and of other authors [12], usually requires the use of the entire mass of the extracted and ground tooth. In this regard, FAAS is more invasive and less useful in population studies. It should be emphasized, however, that in the case of teeth, there are no data indicating the possibility of releasing elements that have been built into their structure in various physiological or pathological situations. Therefore, the elemental composition of the teeth is considered to reflect the cumulative exposure of the body to heavy metals [15].

Unlike teeth, bone tissue in the body is in constant contact with blood and the entire internal environment, contributing to homeostasis. Therefore, the elemental composition of bone may be subject to certain fluctuations related to various physiological and pathological factors influencing the dynamic remodeling of these tissues [16,17]. They also reflect the body’s exposure to heavy metals. On a structural level, there are two types of bone tissue: cortical bone and cancellous bone. It is known that cancellous bone, which has greater blood supply, can provide information on recent exposure to metals, whereas cortical bone may accumulate trace elements over longer periods of time [18,19]. The concentrations of elements in cortical bone are higher than in cancellous bone [20]. In the case of long bones, the concentrations of trace elements are higher in the metaphyseal areas than in their shafts [21]. In toxicological studies, the determination of elements in cortical bone is preferred because it has a more compact structure and is less exposed to contamination resulting from the storage and preparation of samples [22].

Lead and cadmium levels in biological tissues may interact with some essential elements. According to our former research, there are close mutual relationships between lead and iron and between lead and manganese in a whole tooth structure [23]. There are also significant negative correlations between lead and iron, lead and manganese, cadmium and chromium, cadmium and iron, cadmium and manganese and positive correlations between cadmium and copper as well as cadmium and zinc in tooth roots [13]. To the best of our knowledge, there is no information available in the scientific literature on the co-occurrence of lead and cadmium with elements essential in the bone tissue.

A problem with the use of bone tissue in assessing the body’s exposure to metals is its difficult availability. Bone tissue biopsies exclusively for research purposes are not ethically acceptable; therefore, they cannot be considered as a biological material for population biomonitoring of exposure to heavy metals. However, tissues that are waste surgical material generated during surgical operations are available. To date, femoral heads removed during hip replacement [24], knee joint tissues [25], and mandibular cortical bones collected during the removal of impacted wisdom teeth [11] have been used for these purposes. An interesting material in this respect is another craniofacial bone: the maxilla. As a pneumatized bone, it consists mainly of a compact lamina. As a waste surgical material, part of the maxillary bone can be obtained during Caldwell–Luc maxillary sinus surgeries [26]. Currently, the development of less invasive endoscopic methods of maxillary sinus surgery, with the special role of functional endoscopic sinus surgery, has caused Caldwell–Luc operations to be performed relatively rarely [27,28]. The usage of the Caldwell–Luc approach is limited these days and recommended only when better access to the sinus area is needed, for example, when removing large odontogenic cysts, extensive inflammatory lesions, or large foreign bodies [29]. During these procedures, in order to gain surgical access to the maxillary sinus, an opening is made in the anterolateral wall of the maxilla [30]. This bone is irretrievably lost during the procedure. Therefore, by performing the appropriate preparation of the access window, a piece of the maxillary bone can be obtained for research without exposing the patient to excess bone loss. To the best of our knowledge, this research material has never been used before to assess the effects of environmental exposure to heavy metals. The scientific literature also lacks precise data on the content of heavy metals and essential metals in this type of bone tissue.

The aim of the study was to assess whether the elemental composition of the maxillary bone from individuals belonging to a given population reflects the environmental exposure of this population to lead and cadmium.

## 2. Results

Descriptive statistics for the levels of lead, cadmium, iron, manganese, chromium, copper, and zinc in the maxillary bone samples are shown in Table 1. The statistical analysis performed using Student’s *t*-test showed that the lead and cadmium levels in the maxillary bone samples from the residents of Katowice were statistically significantly higher than in the samples from the residents of Bielsko-Biala (the *p*-levels are presented in Table 1). Additionally, the contents of essential metals, i.e., manganese, chromium, copper, and zinc, were higher in the samples from Katowice residents than in the samples from Bielsko-Biala residents. There were no statistically significant differences in the iron levels between the samples from the residents of both cities.

The levels of metals in the maxillary bones, as grouped based on the sex of each of the patients from whom these samples originated, of the inhabitants of Bielsko-Biala are shown in Table 2, whereas those of the residents of Katowice are shown in Table 3. In the samples from the residents of Bielsko-Biala, no significant differences in the content of essential and heavy metals were found between genders (Table 2). On the other hand, the residents of Katowice had higher manganese, chromium, and copper content levels in the samples taken from women than in those taken from men (Table 3).

The metal levels in the samples of the maxillary bones, as grouped based on the distance of each patient’s place of residence from the city center, of the inhabitants of Bielsko-Biala are shown in Table 4, and those of the residents of Katowice are shown in Table 5. Statistical analyses carried out for the residents of both cities using the Mann–Whitney U-test did not show statistically significant differences in the content of seven determined metals based on the distance from the place of residence to the city center (Table 4 and Table 5).

There were also no statistically significant differences between the levels of elements in the samples of the maxillary bones as grouped by educational level in either city (Table 6 and Table 7).

## 3. Discussion

An organism’s environmental exposure to heavy metals can be assessed on the basis of many biological tests, such as tests on the organism’s hair [31,32], nails [33,34], milk teeth [7,35], permanent teeth [13], femoral head [24,25], jaw bone [11], and other parts. To the best of our knowledge, this article is the first research paper to analyze the impact of environmental exposure to heavy metals on the occurrence of heavy metals and essential metals in maxillary bone samples. The intention of this study was to use the surgical waste material in the form of bone blocks taken during the Caldwell–Luc surgical procedure to determine whether the elemental content in this material corresponds to environmental exposure to heavy metals. Taking into account the invasiveness of this surgical intervention, this procedure cannot be routinely used for population biomonitoring.

In our research, it was found that the content of lead and cadmium in samples of the maxillary bone differed statistically significantly between people living in different study areas. The content of lead and cadmium in the samples of the maxillary bones of the inhabitants of Bielsko-Biala was 3.26 ± 2.42 µg/g and 0.74 ± 0.38 µg/g, respectively, whereas in the samples from the inhabitants of Katowice, it was 7.66 ± 2.79 µg/g and 1.12 ± 0.08 µg/g, respectively (Table 1). One of the reasons for this observation may be the significant difference in the content of lead and cadmium in the air in both cities over several years (Table 8). The content of lead and cadmium in PM10 in air samples from Bielsko-Biala in 2006 was 0.027 µg/m^3^ and 0.7 ng/m^3^, respectively, and in 2021, it was 0.006 µg/m^3^ and 0.3 ng/m^3^, respectively. For comparison, the content of lead and cadmium in PM10 in air samples in Katowice in 2006 was 0.062 µg/m^3^ and 1.8 ng/m^3^, respectively, and 0.015 µg/m^3^ and 0.4 ng/m^3^, respectively, in 2021. Earlier studies conducted by other authors confirmed that the content of heavy metals in the head of the femur collected during hip arthroplasty corresponds to the content of these metals in the environment of the place where the patient lives [36]. The content of lead and cadmium in a patient’s hair corresponded to the content of these heavy metals in the environment [37,38]. It has been repeatedly confirmed that the content of lead and cadmium in deciduous and permanent teeth corresponds to the environmental exposures of the people from whom these biological samples were taken [15,39]. The authors of these studies came to the conclusion that these biological samples they use can also be used as materials for conducting environmental biomonitoring. Additionally, in a previously published study by our team on the content of lead and cadmium and selected essential metals in impacted wisdom teeth and the cortical bone of the mandible adjacent to these teeth, it was found that the lead and cadmium levels in these biological samples corresponded with the environmental exposure of the patients from whom these samples were taken [11]. This research further proves that the lead and cadmium levels in the maxillary bone also correspond to the environmental exposures of patients to these heavy metals.

Among the analyzed essential elements, the levels of manganese, chromium, copper, and zinc were also higher in the samples of maxillary bones from the inhabitants of the more polluted area of Katowice (Table 1). This revealed the synergism of the coexistence of metals in calcified tissues, which has been confirmed many times in previous studies [9,40]. This further emphasizes that the accumulation of essential metals in calcified tissues may play a protective role in situations of excessive environmental or occupational exposure of an organism to toxic metals [41,42,43,44,45].

Sex determines the presence of toxic and essential metals in many tissues [46]. This phenomenon is due to many factors, the most important of which is hormonal balance [47]. The authors’ own research showed no statistically significant differences in the presence of lead and cadmium in the samples of the maxillary bones of women and men either in the relatively clean area of Bielsko-Biala or in the more polluted area of Katowice (Table 2 and Table 3). Among the essential metals, higher manganese and chromium content levels were found in the teeth of women from the city of Katowice. The same observations were made in previous studies analyzing the content of these metals in samples of mandibular bones [11]. These differences could be due to different overall bone mineralization between the male and female groups as well as dietary habits, hormone levels, or occupational exposure. However, these factors were not analyzed in the present study.

In order to eliminate the influence of the concentration of industry and motor traffic on the content of the seven determined elements in the samples of maxillary bones, the inhabitants of Bielsko-Biala and Katowice were additionally divided into groups depending on the distance of each patient’s place of residence from their respective city centers (Table 4 and Table 5). Taking into account the urban conditions of both cities, groups living 0–7 km from the city center and 7–14 km from the city center were distinguished. Both in Bielsko-Biala and Katowice, no statistically significant differences were found in the levels of lead and cadmium or the other five measured essential metals in the structure of the maxillary bone. This may indicate that biological tests on maxillary bones reflect the general population’s environmental exposure to metals in their place of residence but are not sensitive to minor modifications to this contamination in the form of local increases of environmental exposure.

Sociological studies emphasize that the level of education may influence one’s health-related behavior, diet, and awareness of the need to protect oneself from environmental exposure, which may also affect exposure to heavy metals [48,49]. Hence, in order to determine whether one’s level of education indirectly influences environmental exposure, which was reflected in the metal content in the samples of maxillary bones, the patients from Bielsko-Biala and Katowice were divided into four groups depending on their level of education (Table 6 and Table 7). It was found that the content of lead, cadmium, iron, manganese, chromium, copper, and zinc did not differ statistically significantly. It can therefore be assumed that exposure to heavy metals of environmental origin is the main determinant of the presence of these elements in samples of maxillary bone.

The original aspects of this study are the type of the biological material used and the measuring of five essential metals in addition to lead and cadmium. Although we tried to exclude the effects of exposure to heavy metals other than air, we assessed them on the basis of the patient’s declarations about leaving their place of residence long-term, smoking cigarettes, taking medications and dietary supplements, and eating store-bought rather than home-grown food. However, the impact of these factors on the content of metals in the maxillary bone cannot be unequivocally ruled out. Although the patients from whom the maxillary bone samples were taken during the Caldwell–Luc operations were divided according to the distance of their place of residence from the city center, it is impossible to determine over the years how often and for how long they moved between these zones.

This is undoubtedly a limitation of this research work. Moreover, the fact that the research material took the form of maxillary bones collected during quite invasive Caldwell–Luc maxillary sinus surgery limits the availability of this material and makes it impossible to be used in population biomonitoring.

## 4. Materials and Methods

The research material consisted of cortical bone from the anterolateral maxillary wall collected from 126 patients during Caldwell–Luc maxillary sinus surgery due to chronic sinusitis or the presence of cysts. All of the samples were from patients aged 18 to 40 years (mean age 32.8 ± 8.2 years). In all cases, only one sample was from one patient. The samples were collected from 64 residents of Bielsko-Biala (42 women and 22 men) and 62 residents of Katowice (39 women and 23 men). The choice of the cities from which the patients came was intentional. The cities are 50 km apart and have completely different levels of environmental exposure to heavy metals [50].

Katowice, which is inhabited by over 265,000 people, is the capital of the Upper Silesia industrial district, where concentrations of heavy metals in the air have been high for many years [51]. Despite the restructuring processes that have been undertaken over the past 30 years, heavy industry still shapes the economy of this region and pollutes the natural environment. Bielsko-Biala, which has a population of over 170,000 inhabitants, is often referred to as “the capital of Podbeskidzie”. Bielsko-Biala is a tourist region and has a relatively clean natural environment. The main source of pollution in this region is so-called “low-emission” and long-distance pollution from the Czech Republic and Slovakia [51]. Considering the mean age of the patients from which the maxillary bone samples were derived (32.8 ± 9.6 years) and the fact that neither the period of metal accumulation in the maxillary cortical bone nor the environmental pollution data at the time of the study were known, the authors took into account historical data. Therefore, Table 8 contains data on the content of PM 10 in the air and the content of lead and cadmium the in PM 10 in the years 2006 to 2021 at 5-year intervals. These data clearly show that the lead and cadmium contents in the air in Katowice were consistently higher than the contents of these heavy metals in the air in Bielsko-Biala over this period of 15 years. It should be noted here that air quality is considered to be the most important factor representing the total pollution of the environment and to have the greatest impact on the health status of the population living in a given area [52]. An important aspect of our research project is the fact that the supply of tap water to both Katowice and Bielsko-Biala comes from one water supply system, the central point of which is the Goczalkowice reservoir [53]. However, this assumption might be imperfect, as a significant amount of lead exposure in drinking water has been found to be due to local sources, such as lead service lines and lead-containing plumbing [54,55]. Therefore, without individual data on lead in drinking water, this comparison should be considered an indirect contribution to lead exposure from drinking water.

In our research, we decided to also determine, apart from lead and cadmium, the content of such essential metals as iron, manganese, chromium, copper, and zinc. We took into account that their concentrations in the maxillary bone may change as a result of interactions between toxic and essential elements.

Taking into account the data collected in Table 8 and the information provided above, the selection of the cities from which patients came guaranteed a different degree of environmental exposure to cadmium and lead.

All patients from whom maxillary bone samples were collected for research declared that they had lived in Katowice or Bielsko-Biala since birth and had not left the area of these cities for a period longer than 2 months. Moreover, all patients declared that they did not smoke cigarettes, were not exposed to tobacco smoke in their apartments, and that they did not take any medications or dietary supplements on a permanent basis. They also declared that they were not on any special diets and that the food they ate came from shops and not from home crops. The preoperative laboratory diagnostics performed included blood counts, coagulation parameters, and peripheral blood ionograms. Laboratory tests and pre-operative interviews with the maxillofacial surgeon and anesthesiologist showed no abnormalities in any of the patients.

In order to find out whether motor traffic in the city center affects the metal levels of the samples of the maxillary bones, patients were divided into two groups according to the distance from their place of residence to the city center: from 0 to 7 km from the city center (38 patients from Bielsko-Biala and 41 patients from Katowice) and from 7 to 14 km from the city center (26 patients from Bielsko-Biala and 21 patients from Katowice).

The patients were also divided into four groups depending on their level of education: primary education (8 patients from Bielsko-Biala and 11 patients from Katowice), vocational education (24 patients from Bielsko-Biala and 29 patients from Katowice), secondary education (15 patients from Bielsko-Biala and 14 patients from Katowice), and higher education (17 patients from Bielsko-Biala and 8 patients from Katowice).

**Figure 1:**
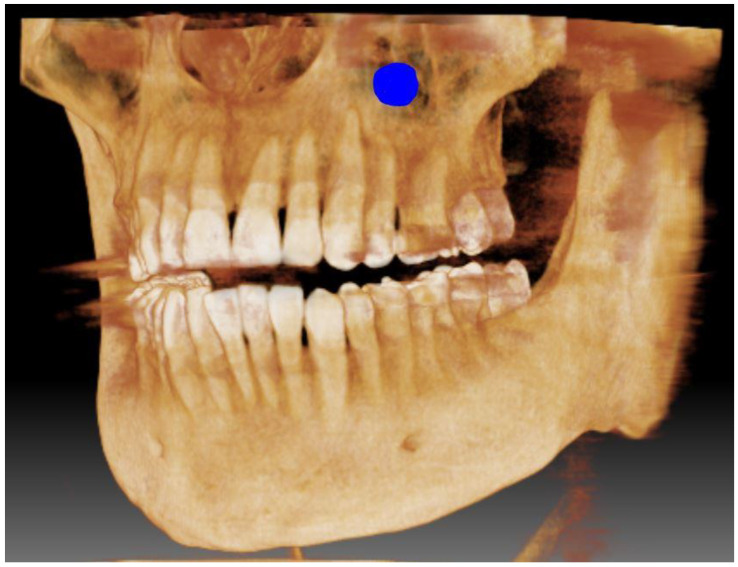
The approximate location of the bone sample on the anterolateral wall of the maxilla (marked in blue).

All samples were collected by the same specialist maxillofacial surgeon (P.M.) during Caldwell–Luc maxillary sinus surgery performed under general anesthesia due to chronic inflammatory processes in the maxillofacial sinuses or sinus cysts. After incision of the mucoperiosteal flap in the atrium of the oral cavity, the anterolateral wall of the maxilla was exposed. Using a piezo-surgical unit (Piezotome Cube F50100, Acteon Group Ltd., Norwich, UK), a bone window was excised with dimensions corresponding to a standard Caldwell–Luc surgical access opening (Figure 1). Immediately after excision, the bone window was rinsed copiously with double-distilled water and placed in a sealed Teflon container where it was stored until further preparation. The samples were further prepared according to the protocol described previously for chromium [14]. Bone specimens were dried to constant weight at 105 °C and then pulverized and stored in a desiccator. All of the samples were re-dried at 105 °C for 4 h immediately before dissolution. Samples weighing from 0.200 to 0.500 g of dry weight were dissolved in 3 mL of ultrapure concentrated nitric acid (Supra-Merck 65%, Merck, Darmstadt, Germany) in a Teflon vessel for 3 h at 120 °C. The resulting clear solution was then diluted to 10 mL with double-distilled water.

The lead, cadmium, iron, manganese, chromium, copper, and zinc concentrations in the sample solutions were determined with flame atomization atomic spectrometry using a Philips PYE Unicam SP-9 instrument (Philips, Amsterdam, The Netherlands). An air–acetylene flame was used in the markings. The absorption lines used for lead, cadmium, iron, manganese, chromium, copper, and zinc were 283.3, 228.8, 248.3, 279.5, 357.9, 324.8, and 231.9 nm, respectively. The detection limits set in our laboratory tests for lead, cadmium, iron, manganese, chromium, copper, and zinc were 0.025, 0.018, 0.090, 0.044, 0.075, 0.055, 0.020 mg/L, respectively.

The study used a certified bone meal reference material (SRM 1486; US National Institute of Standards and Technology, Gaithersburg, MD, USA) for cadmium, copper, iron, lead, manganese, and zinc. For chromium, a certified bone ash reference material (SRM 1400; US National Institute of Standards and Technology, Gaithersburg, MD, USA) was analyzed. The certified concentrations and metal levels determined in our research are presented in Table 9. The precision of the applied method was close to 8%, whereas the accuracies for different elements ranged from 3.01% to 6.99%.

### Statistical Analysis

In order to carry out a statistical analysis, the data were first grouped according to the place of residence of the patient from whom the samples came. The Kolmogorov–Smirnov goodness-of-fit test and observations of the distribution plots showed that the data were not normally distributed. Therefore, we subjected the data to a natural logarithmic transformation, and the data distribution became normal. This allowed for the use of Student’s *t*-test to determine the significance of the differences in the concentrations of elements between the samples from the residents of Bielsko-Biala and Katowice. The same data-conditioning procedure has been used in previous studies [23,39,56].

Due to the fact that the number of the samples collected from men and women differed significantly, the non-parametric Mann–Whitney U test was used to assess the significance of the differences between the levels of elements in the maxillary bone samples. The non-parametric Mann–Whitney *U* test was also used to analyze the significance of the differences between the contents of elements in the maxillary bone depending on the distance from the patient’s place of residence to the city center. The non-parametric Kruskal–Wallis test was used to assess the significance of the differences between the concentrations of elements in the samples of the jaw bone depending on the patient’s education (primary, vocational, secondary, and higher education). *p* < 0.05 was considered statistically significant. Statistical analyses were performed using Statistica 13.1 software (Statsoft, Tulsa, OK, USA).

## 5. Conclusions

The levels of lead and cadmium in the maxillary bone corresponds to the environmental exposure to these heavy metals in the patient’s place of residence, which was proven in this example of residents of two cities with different concentrations of these heavy metals in the air over long time periods. Additionally, higher levels of essential metals such as manganese, chromium, copper, and iron are characteristic of the maxillary bone samples of the inhabitants of an area that is more polluted with heavy metals. Due to the invasiveness of the surgical procedure, the maxillary bone samples cannot be used for routine biomonitoring or for research purposes only. However, in very specific situations where patients would be qualified for Caldwell-Luc surgery because of particular medical indications, the maxillary bone samples as surgical waste material could be used in population biomonitoring of exposure to heavy metals. In order to use maxillary bone samples for this purpose, further research is needed to eliminate other factors that may affect their elemental composition.

## Figures and Tables

**Table 1:** Descriptive statistics for the heavy metal concentrations (µg/g) in the maxillary bones of people living in Bielsko-Biala and Katowice.

	Samples from People Living in Bielsko-Biala (*n* = 64)			Samples from People Living in Katowice (*n* = 62)			
Element	Mean ± SD	Min–Max	95% Limit of Confidence	Mean ± SD	Min–Max	95% Limit of Confidence	*p*-Level *
Pb	3.26 ± 2.42	1.24–7.13	2.65–4.95	7.66 ± 2.79	2.12–14.92	6.22–9.12	<0.001 ^a^
Cd	0.74 ± 0.38	0.12–1.86	0.42–1.12	1.12 ± 0.08	0.11–2.87	0.98–1.62	0.007 ^a^
Fe	21.54 ± 11.44	5.66–49.12	15.44–28.16	18.22 ± 10.74	3.12–46.79	14.27–21.98	0.652
Mn	1.76 ± 1.11	0.14–3.87	1.12–2.47	4.87 ± 2.22	0.27–8.12	3.12–5.54	<0.001 ^a^
Cr	1.82 ± 1.62	0.22–4.12	1.16–2.53	3.96 ± 4.12	0.14–15.69	2.56–5.78	0.022 ^a^
Cu	7.12 ± 4.08	1.66–14.29	5.14–10.11	14.27 ± 7.72	4.12–30.16	11.77–16.87	<0.001 ^a^
Zn	149.12 ± 44.29	63.27–278.66	98.52–211.44	246.13 ± 108.92	82.45–412.87	187.45–301.24	<0.001 ^a^

*n* = number of samples. * *p*-levels, which were determined using Student’s *t*-tests, were used to identify statistically significant differences between the mean heavy metal concentrations in the samples provided by people living in Bielsko-Biala and Katowice. ^a^ indicates where the heavy metal concentrations in the samples provided by people living in Bielsko-Biala and Katowice were significantly different.

**Table 2:** Heavy metal concentrations (µg/g) in the maxillary bone samples provided by women and men living in Bielsko-Biala.

Element	Women (*n* = 42) Median	Men (*n* = 22) Median	*U*-Value	*p*-Level *
Pb	4.12	3.06	92	0.326
Cd	0.81	0.69	87	0.388
Fe	19.18	26.32	98	0.422
Mn	1.94	1.52	79	0.343
Cr	2.02	1.66	84	0.254
Cu	8.11	5.89	88	0.366
Zn	152.75	144.54	103	0.720

*n* = number of samples. * *p*-levels, which were estimated using the Mann–Whitney U test, were used to identify statistically significant differences between the metal concentrations in the samples provided by women and men.

**Table 3:** Heavy metal concentrations (µg/g) in the maxillary bone samples provided by women and men living in Katowice.

Element	Women (*n* = 39) Median	Men (*n* = 23) Median	U-Value	*p*-Level *
Pb	8.94	6.82	96	0.226
Cd	1.33	1.08	101	0.824
Fe	16.89	21.24	98	0.351
Mn	5.69	3.86	54	0.022 ^a^
Cr	6.62	1.54	51	0.008 ^a^
Cu	18.42	9.06	48	0.048
Zn	273.12	202.33	78	0.062

*n* = number of samples. * *p*-levels, which were estimated using the Mann–Whitney U test, were used to identify statistically significant differences between the metal concentrations in the samples provided by women and men. ^a^ indicates that the heavy metal concentrations in the samples provided by women and men were significantly different.

**Table 4:** Heavy metal concentrations (µg/g) in the maxillary bones of people living at different distances from the center of Bielsko-Biala.

Element	0–7 km (*n* = 38) Median	7–14 km (*n* = 26) Median	U-Value	*p*-Level *
Pb	2.98	3.42	112	0.122
Cd	0.72	0.81	92	0.336
Fe	22.78	19.84	84	0.082
Mn	1.62	1.84	102	0.523
Cr	1.66	1.89	113	0.425
Cu	7.28	6.92	99	0.226
Zn	167.23	131.26	113	0.092

*n* = number of samples. * *p*-levels, which were estimated using the Mann–Whitney U test, were used to identify statistically significant differences between the metal concentrations in the samples provided by people living at different distances from the center of Bielsko-Biala.

**Table 5:** Heavy metal concentrations (µg/g) in the maxillary bone from people living at different distances from the center of Katowice.

Element	0–7 km (*n* = 41) Median	7–14 km (*n* = 21) Median	U-Value	*p*-Level *
Pb	7.92	7.24	101	0.229
Cd	1.64	0.97	89	0.663
Fe	16.37	19.44	92	0.811
Mn	4.12	5.29	76	0.219
Cr	3.62	4.12	91	0.118
Cu	13.22	15.01	78	0.328
Zn	231.22	278.19	81	0.691

*n* = number of samples. * *p*-levels, which were estimated using the Mann–Whitney U test, were used to identify statistically significant differences between the metal concentrations in the samples provided by people living at different distances from the center of Katowice.

**Table 6:** Heavy metal concentrations (µg/g) in the maxillary bones of people with different levels of education living in Bielsko-Biala.

	Educational Level					
Element	Primary (*n* = 8) Median	Vocational (*n* = 24) Median	Secondary (*n* = 15) Median	Higher (*n* = 17) Median	*H*-Value	*p*-Level *
Pb	3.44	3.12	3.98	2.98	1.924	0.892
Cd	0.82	0.65	0.72	0.77	3.882	0.587
Fe	22.65	28.54	21.56	18.74	4.341	0.645
Mn	1.66	1.94	1.22	1.12	3.226	0.447
Cr	1.64	2.02	1.93	1.77	2.998	0.682
Cu	6.89	8.99	6.12	7.01	3.882	0.221
Zn	162.66	127.55	140.29	152.89	2.669	0.442

*n* = number of samples. * *p*-levels, which were estimated using the ANOVA Kruskal–Wallis test, were used to identify statistically significant differences between the metal concentrations in the samples provided by people with different levels of education living in Bielsko-Biala.

**Table 7:** Heavy metal concentrations (µg/g) in the maxillary bones of people with different levels of education living in Katowice.

	Educational Level					
Element	Primary (*n* = 11) Median	Vocational (*n* = 29) Median	Secondary (*n* = 14) Median	Higher (*n* = 8) Median	*H*-Value	*p*-Level *
Pb	8.12	8.44	7.12	6.82	4.101	0.367
Cd	1.42	1.62	0.88	1.01	2.522	0.622
Fe	17.77	16.22	17.98	19.43	3.112	0.882
Mn	4.92	5.11	3.78	4.91	2.117	0.229
Cr	4.12	3.11	3.87	4.22	1.119	0.691
Cu	12.29	17.24	14.29	14.87	3.972	0.711
Zn	188.11	192.88	297.12	289.14	4.008	0.311

*n* = number of samples. * *p*-levels, which were estimated using the ANOVA Kruskal–Wallis test, were used to identify statistically significant differences between the metal concentrations in the samples provided by people with different levels of education living in Katowice.

**Table 8:** The average annual PM 10, Cd in PM 10, and Pb concentrations in the air in Bielsko-Biala and Katowice.

Year		Pb in PM 10 [µg/m^3^]	Cd in PM 10 [ng/m^3^]	PM 10 [µg/m^3^]
2006	Bielsko-Biala	0.027	0.7	35.7
	Katowice	0.062	1.8	58.4
2011	Bielsko-Biala	0.023	0.9	43.3
	Katowice	0.051	1.9	49.9
2016	Bielsko-Biala	0.026	1.0	35.8
	Katowice	0.060	1.8	38.6
2021	Bielsko-Biala	0.006	0.3	25.6
	Katowice	0.015	0.4	32.2

Source: Data obtained from the database of the General Inspectorate for Environmental Protection in Katowice (Glowny Inspektorat Ochrony Srodowiska) http://powietrze.gios.gov.pl (accessed on: 9 September 2022).

**Table 9:** Certified and measured concentrations of heavy metals in the reference materials (µg/g).

Element	Certified Value	Experimental Value
Pb	1.335 ± 0.014	1.483 ± 0.024
Cd	0.011 ^a^	0.013 ± 0.010
Fe	99 ± 8	112 ± 14
Mn	1.16 ^a^	1.32 ± 0.36
Cr	0.03 ^b^	0.03 ± 0.010
Cu	0.8 ^a^	0.91 ± 0.07
Zn	147 ± 16	141 ± 12

SRM 1486 bone meal (Cd, Cu, Fe, Mn, Pb, and Zn concentrations) and SRM 1400 bone ash (Cr concentrations) were used; both reference materials were supplied by the US National Institute of Standards and Technology (Gaithersburg, MD, USA). ^a^ Non-certified value. ^b^ SRM 1400 bone ash; non-certified value.

## Data Availability

The data presented in this study are available on request from the corresponding author.
